# Color Matching Generation Algorithm for Animation Characters Based on Convolutional Neural Network

**DOI:** 10.1155/2022/3146488

**Published:** 2022-08-20

**Authors:** Jiali Lyu, Hae Young Lee, Huwen Liu

**Affiliations:** Cheongju University, Cheongju 28503, Republic of Korea

## Abstract

In recent years, for China, animation industry is a relatively new and mature emerging national sunrise industry after animation industry, which appears on the world stage more and more frequently and is widely concerned and valued by people from all over the world. Therefore, this paper innovatively uses the convolutional neural network algorithm to innovate the color matching generation of animation characters and improve the traditional technology of color matching for animation characters. In this paper, we mainly use Generative Adversarial Network (GAN), Deep Convolutional Generative Adversarial Network and VGG model, and multiscale discriminator theory and use ACGAN research method. And we study this paper's innovative LMV-ACGAN research method, and we have come to the conclusion that other models have higher collapse rate than this model; this model has higher color matching of anime characters. Color matching improves with the increase of convolutional neural network utilization, etc. Moreover, superior and minor reviews of this study are provided to make later researchers understand this study more rationally and objectively.

## 1. Introduction

Animation production is another contemporary Chinese specialty that can be completely said to integrate ancient Chinese excellent literature, film, photography, painting, music technology, etc. into one large modern film and television integrated animation production and art field talents [1]. In the early 1960s and 1970s, the animation industry itself was a new high-tech sunrise industry created by another developed country like Japan, and it has been receiving more and more attention from governmental agencies all over the world, and in the future, it will be more and more common and deep and will be used in extensively commercial applications in all walks of life around the world. In today's general trend that the market and competition in the field of animation in China and the world are developing more and more intensively, exploring and summing up such a correct development path, which is suitable for the development of the new mode of China's animation culture industry today and in the future, have become one of the primary issues that we have to pay serious attention to and consider at present, as well as every Chinese animation person who is concerned about China's problems. It has become a primary issue that we have to pay serious attention to and take into consideration. Fortunately, after the release of a series of excellent foreign anime series such as “Mulan” series and “Kung Fu Panda” series, many Japanese anime designers who like Chinese works have slowly started to realize that the traditional Chinese art culture of our ancient times is a rich and profound source of knowledge. The traditional Chinese art culture has created a rich variety of design and creation materials for us [2].

The design of anime character modeling is one of the most fundamental cores of designing all excellent anime works. The design methods of anime character modeling usually include the design of anime character modeling features, character color features, clothing style features, and action modeling features. The color part is another important visual symbol to show the most direct, vivid, and intuitive character effect in anime, and the matching use of color elements and the design and application of color generation and effect have always occupied a significant position in almost all the design methods of anime character modeling [3]. Modern psychological research and development has long told us that about 80% of children's main cognitive and psychological sources of information and their main social and behavioral power sources are either directly or indirectly from or rely on visuals, so for every normal child like us, a quick glance as a child will, therefore, make it clear what we see, and later, if we look again as we grow up, we may understand how to gradually absorb what is there and imitate what is being created [4]. Academics generally believe that our country should strongly guide and advocate Chinese animation talents in practice to create and design a series of excellent Chinese animation characters with distinctive Chinese national culture, spiritual, and cultural characteristics connotation, and we should take this as an important measure to promote the formation of an animation characteristic cultural strategy for enterprises to actively explore the formation of enterprises with their own individual unique value of the animation market [5].

With the rapid development and popularization of computer technology and technological progress in the information age, algorithms have increasingly become an essential digital technology applied in people's daily work production and practical life [6]. Because this algorithm technology itself has another kind of application scene with strong and highly consistent interactive characteristics and deep learning autonomy, it is more widely and effectively used in many related industry fields such as medical simulation surgery, entertainment game interactive experience, decorative home environment optimization design, animation scene game design, and production process [7]. The scale of digital information technology product application tends to be increasingly popularized and developed at a high speed, which greatly leads to and effectively promotes the smooth and healthy development of the overall pattern of the world domestic film and animation business, which will be regarded as an indispensable and part of the coordinated development of the whole digital culture field industry chain system in China today and has become more and more a part of our promotion of national social and economic and cultural prosperity and global economic and social development, that is, the most important spiritual impetus for the harmonious and simultaneous promotion of national social and cultural prosperity and global economic and social development [8]. In order to further promote the rapid and comprehensive development of the whole and even China's animation industry, through further rationalization and optimization of algorithm technology and application, a set of more perfect and feasible animation character designs and workflows can be quickly formed, which can quickly determine a specific and accurate 3D animation character according to the actual needs of animation character design. It can determine a specific and accurate three-dimensional animation character and can quickly carry out collision trajectory and detection and analysis, so that each animation character can automatically and accurately detect and calculate the most accurate and reasonable collision flight path and can quickly and automatically complete the automatic tracking conversion of target tracking, flight trajectory tracking, and other action mode trajectories, so as to achieve the perfect animation character in the combination of simulation and reality. The perfect roaming in the animation scene system is required [9].

## 2. Research Background

To match the color of anime characters, let us first understand the definition of manga and animation separately. What is manga? The word “manga” in manga refers to the illustration of the water in the tank beyond the mouth of the tank. This description serves as a representation of the essence of the painting. It means that the character of the painting is the same as the water coming out of the tank. It is a description that goes beyond the description of the current situation. It is also a statement of supernatural expression. Therefore, the character cartoons appearing in the press are representatives of simple and easy-to-understand cartoons. These cartoons usually exaggerate and mutate the drawn objects, magnifying the character's characteristics without losing his nature, depicting life in a simple and exaggerated way, reflecting reality, and generating a sense of fun at the same time. This is how good works usually cause us to have a laugh and resonate with each other [10].

In terms of the development process, animation is the brother of manga, and the creation of animation is usually accompanied by a good manga script. The realization of animation is, in short, the coherent piecing together of a single comic work. Like the animated miniature books that we saw as children, they are composed of many dynamically connected and progressively developing pictures, which are quickly flipped through to obtain a vivid visual effect. It can also be said that animation is a moving comic strip, a combination of film and painting art. Long before digital technology was created, people already began to use simple, crude methods to create animation. Although the effect is far less than nowadays, the brilliant achievements of modern animation industry are inseparable from such exploration [11].

Animation is based on cartoons. As an extension of cartoons, animation automatically inherits the expressive function of cartoons. This means of expression can be used to make up for the lack of performance and expression of real people and objects, especially when it comes to exaggerated and distorted shots or magnificent scenes. From the production method, “animation” is a kind of video technology; that is to say, whether the traditional production of hand-drawn animation, or hand-drawn and computer coloring, and other technical means of combining the new form of animation basically relies on computer animation (including 3D and 2D), it is the use of technical means to achieve the video performance mode. In terms of expression, animation is a mode of running, from the moment of its birth, the way of its picture movement determines that it must be built based on traditional painting, film, music, theater, and other artistic expressions before seeking development. The subversion of live action and traditional theater in animation has also reached new heights with the prevalence of 3D technology today [12].

The specific classification of animation does not have any relatively fixed standard regulations. From the traditional drawing and production of animation, which is mainly classified according to the technical principle and modern art methods, animation art can be roughly divided into most of the traditional mechanical hand-drawn works, which are mainly based on the basic expression technology, and a few works, which are mainly based on the direct use of computer software to draw the basic content form. Mechanical computer animation: according to the closest to its television closest to the natural realistic artistic movement form method of these two kinds of animation of the basic action of the artistic effect expression form method and the basic action expression form method to separate and then according to be relatively comparable to the specific countries and regions, animation television can be divided according to the action of roughly close to the degree of reality and can be roughly divided into the action of the closest to the animation television closest to the natural realistic artistic action expression method of animation TV “perfect animation” (animation TV), and action is relatively more than animation TV to simplify some exaggerated action effect of TV animation TV such as “limited animation” (slide animation), and so on. If we can just move simply from the three-dimensional space plane out of the three-dimensional plane visual effect form and hierarchical relationship to cut, the roughly visual picture can further be cut into three-dimensional and flat animation (such as “cat and mouse”) two-dimensional cartoon and three-dimensional animation (such as “monster Shrek”). Making a beautiful and high quality full animated film is a very complex process, while making a semianimated film is relatively simple. But in fact, no matter what kind of animated film it is, it must not be like the old live-action animated film, which first shot a lot of film, and the actors can of course only go to the temporary to play their own style characteristics, in the postediting process to try to cut out the extra parts they do not need to cut and add some relevant sound elements and background music, or even reshoot and cut into the film. The film can even be reshot and reedited into the film. In the process of animation production and implementation, we need to be able to accurately plan and control the specific time and number of images needed to complete each action in advance, and to ensure that there will not be any extra time images during the implementation process, so as to avoid some serious waste of financial resources, energy, and time.

In this paper, based on the above background analysis, we use algorithm technology, especially convolutional network technology, to study anime characters, especially their color matching generation algorithm, thus to innovate the color matching technology of anime characters and contribute to the improvement of our animation technology.

## 3. Research Method and Basic Theory

### 3.1. Basic Theory

#### 3.1.1. Generative Adversarial Network (GAN)

Generative adversarial network is a generative model proposed by Dellowgeed in 2004. The core idea of the model is “zero-sum game” thinking, and the more important is the optimization objective function, as shown in the following formula:

(1) $$\begin{array}{c} \underset{G}{\text{min}}\underset{D}{\text{max}}V\left(D,G\right)=E_{X∼P_{\text{data}}}\left(x\right)\left[\text{log}D\left(X\right)\right]+E_{Z∼P_{\text{data}}}\left(Z\right)\left[\text{log}\left(1-D\left(X\right)\right)\right], \end{array}$$

where *z* is the relevant noise value, from the *P*_*z*_ distribution of the collected samples, to generate the *G* latest required network, and *Z* according to the *G*_*z*_ formed is thus derived, while giving the color image 0 a label ““, meaning that the generated one is a false image. At the same time as that the false image, the true *P*_data_ image is read from the *x* relatively correct distribution, and a code ““ is given to 1. Subsequently, *G*_*z*_ is fed to the discriminator *D* together with *x* for discriminating. For the discriminator *D* that is used to discriminate, the value of *D*_*G*(*Z*)_ is close to 0, while the value of *D*_*X*_ is close to 1; and with the true and false images, the value of the discriminator is used to reflect *D*, given *G* with to update the parameters [13].

#### 3.1.2. Deep Convolutional Generative Adversarial Network with VGG Model

Convolutional neural networks may have been originally designed or developed specifically to deal with supervised object-oriented machine learning problems, but they have also evolved to include a series of unsupervised object-oriented machine learning system paradigms, including Convolutional Auto Encoders (CAE), Convolutional Restricted Boltzmann Machine (CRBM), Deep Convolutional Generative Deep Confidence Adversarial Machine (CRBM), and Deep Convolutional Generative Deep Confidence Adversarial Machine ((CRBM), CAE), Convolutional Restricted Boltzmann Machines (CRBM)/Deep Convolutional Deep Belief Adversarial Networks (CDBN), and Deep Convolutional Generation Deep Convolutional Generative Adversarial Networks (DCGAN). These hybrid learning algorithms can generally be directly considered as another hybrid learning algorithm constructed by introducing deep convolutional neural networks in another original version of the unsupervised learning algorithm. Therefore, the Deep Convolutional Adversarial Generative Network (DCGAN), which we focus on in this chapter, is one of the core fundamental components of DCGAN.

The deep convolutional adversarial supervised generative network model (DCGAN) was first proposed by Radford et al.'s team in 2016. This network model is also the most effective combination of a deep convolutional neural network system (CNN) network model and a GAN network model and is based on another label-free, nonadversarial supervised generation network model. The two adversarial network models generated by the deep convolutional adversarial network model are actually replaced by the other two adversarial network models, the *G* model and the *D* model of the other model generated by the deep convolutional adversarial network model, and at the same time, the work required to do these two network models is replaced by the other two full-depth convolutional neural network models. The two models are replaced by two other full-depth convolutional neural network models, and at the same time, some specific technical improvements are made to make the whole adversarial network model into another full-depth convolutional neural network.

The VGG model group (Oxford Visual Geometry Group) was originally used as a model for image feature classification systems, but the convolutional neural network part of the model has since been proven by scientists to provide a good technical solution for image feature classification extraction. Technical improvements, which can be used to extract more complete and rich image features with greater visual discrimination [14].

**Figure 1:**
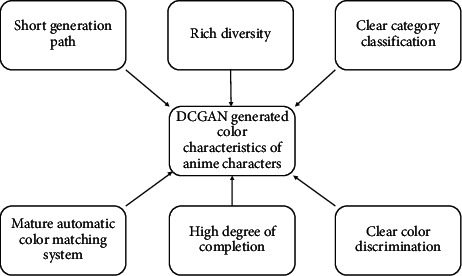
Color characteristics of anime characters generated by using DCGAN.

The color matching generation computation and confrontation network formed based on such network structure has been basically realized that it can be directly used in computers for various anime character color matching generation computation and generation, and although there are more problems, it still has the following six characteristics, that is, short generation path, rich diversity, clear type classification, mature automatic color matching system, high degree of completion, and clear color discrimination, as shown in Figure 1.

#### 3.1.3. Multiscale Discriminator

**Figure 2:**
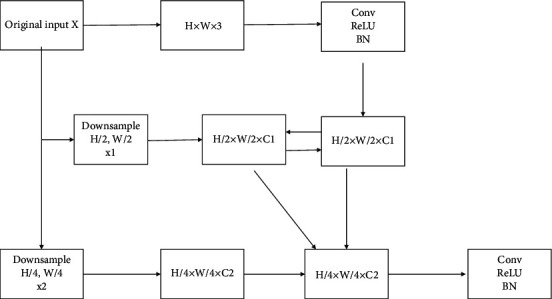
Multiscale feature fusion.

The multisample image scale discriminator technology generally refers to the system that can perform random sampling operation from any one of the images, or at most several times, to obtain one more image of different image sizes and input the image information of all these different image sizes to the corresponding image type of the image information obtained from all these different image sizes that are input to the discriminator system (convolutional neural network) of the corresponding image type to perform the fast extraction and recognition operation of the relevant image feature signals and to complete the operation of various image subsequent recognition related operations. The size feature map of the size feature map of different size images can be built on the base map of the spatial position of each size feature in the range of the size discriminator in the network area map and can be converted into the size feature map of the next feature by convolution, and the size feature map of the size feature map containing a size feature on the base of the area map can be extracted from it. This network approach is also a network approach that fuses the information of scale objects and feature maps to identify and extract information by fusing multiple scale information in the network. The fusion of information is performed, and the final result is to be able to obtain the feature information of the identified object or the identified feature result of the object to be discriminated by the extracted object feature map [15]. This is shown in Figure 2.

#### 3.1.4. Color Matching of Anime Characters

Anime culture refers to a game or a form of entertainment and leisure activity that is currently accepted by the largest masses of human beings and is a product of social reality in a modern and informationized society where human beings are actively seeking to obtain a certain cultural and spiritual value of liberation and a kind of spiritual and aesthetic support characteristics and its realistic spiritual orientation value. The design ability of anime character is one of the most core and key signs to evaluate whether the final form of a domestic original game and animation work can really obtain commercial success. Contemporary Chinese social and cultural life is in a new era of map reading, and symbolized textual information, pictorial information, and dynamic body movement information are undoubtedly among the most popular and easy-to-understand ways accepting new information forms found in the current contemporary environment. The visual image of the animation character is also a visual symbol that reflects its own culture and its aesthetic and cultural appreciation function, which plays an extremely important role in visual culture and aesthetic cognition [16].

The Chinese society in China has a relatively long history and many members and has many excellent cultural traditions and cultural resources that are much better than those of China. Since the development of more than 2.5 centuries, these elements of the world's traditional art have been perfectly integrated into the excellent original cartoon series designed by many famous original cartoon designers in the world today, so that they can be shown in a more unique and vivid form in a more original and unique expression. However, these excellent classic cartoon works are often not really able to fully and skillfully utilize these excellent classic elements. The main drawback of these classic animation works is that they only use a small part of the traditional Chinese costumes and patterns and do not use the traditional Chinese color matching and animation generation methods to use the traditional Chinese elements in a deeper way, animation elements. As a new generation of Chinese animation workers, we need to quietly continue to study, analyze, research, and organize the material culture of the Chinese nation with thousands of years of excellent national history and traditions, so that the most ancient and valuable historical material cultural assets of our nation can be truly preserved and carried forward in a better way. The research results of the project also focus on the in-depth analysis and systematic research to establish the theory of color matching design and the combined color design technology method of our traditional animation characters, which will be of great importance and innovation for the inheritance and continuation of the design of our national and traditional animation national cultural characteristics and for the continued research and development of the modern Chinese traditional color matching technology method of animation characters. This paper uses a variety of research methods to study the significance of this research.

Therefore, this paper uses various research methods to study the generation algorithm of color matching for anime characters, in order to provide reference for anime designers and help the anime industry to add more Chinese traditional cultural factors and promote the development of China's anime industry.

### 3.2. Research Methods

#### 3.2.1. ACGAN Research Method

ACGAN is an image generation model designed by European computer scientist Odena et al. before 2017 that can autonomously generate images according to the conditions autonomously and, at the same time, improve the quality of automatic image generation. For the training sample set, this model requires not only the sample images, but also the category labels corresponding to each training sample [17].

The loss function simulated by this model mainly consists of the following two parts: one is the loss caused by judging the authenticity of pictures; the other is the loss caused by classifying pictures using discriminators. The specific form of the loss function is shown as follows:

(2) $$\begin{array}{c} L_{s}=E\left[\text{log}P\left(\frac{S=\text{real}}{X_{\text{real}}}\right)\right]+E\left[\text{log}P\left(\frac{S=\text{real}}{X_{\text{real}}}\right)\right], \end{array}$$

(3) $$\begin{array}{c} L_{s}=E\left[\text{log}P\left(\frac{C=c}{X_{\text{real}}}\right)\right]+E\left[\text{log}P\left(\frac{C=c}{X_{\text{real}}}\right)\right]. \end{array}$$

Among them, *L*_*s*_ is the loss caused by discrimination of true and false *L*_*c*_ images loss caused by map classification, *X*_fake_=*G*(*z*, *e*) are the generated images: training *X*_real_ images: discrimination of *P*(*S*/*X*) truth and falsehood: *P*(*C*/*X*) classification discrimination.

Because the two loss functions are logarithmic likelihood functions, which are of the same magnitude as *L*_*c*_+*L*_*s*_ numerically, they will maximize *D* to *L*_*c*_ − *L*_*s*_ test and maximize *G* to test.

#### 3.2.2. LMV-ACGAN Method

This article was to create model by using convolution based on neural network part of discriminant by further expanding it into a set of multidimensional scale information, which can help the authors and readers in the process of building model with detailed information and the comprehensive characteristics of some objective feeling, also revised structure of VGG better adapted to the actual task. Finally, the variable tag is applied to add the structure function, and the task of avoiding model collapse is completed well. A huge amount of experimental data proves that the model in this paper plays a good role in animation color matching [17].

**Figure 3:**
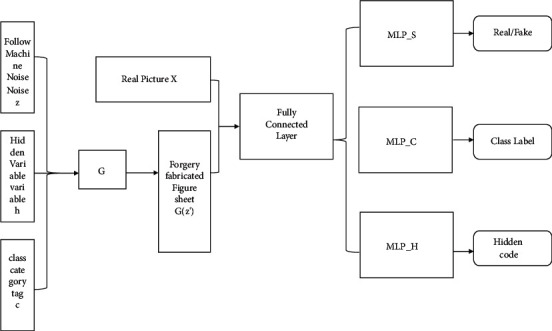
Overall structure of LMV-ACGAN model.

LMV-ACGAN model has a good structure. The input variables of this model are mainly composed of *z* three parts: noise hidden *h*, parameter variable *c*, and label. The author uses vector *z* to *h* synthesize, and *c* and *z* input the *G* generator to generate the image color through the convolutional neural network to get the *G*(*z*') fake image. At the same time, the *x* true *G*(*z*') image and the common image are sent to D_CNN, and the convolution neural network is used for flatten operation to make the feature graph into a one-dimensional feature vector, and the changed feature vector is input to the following three MLP_S, MLP_C, and MLP_H, respectively, to judge the true and false images, classify images, and calculate printing parameter values. The overall structure is shown in Figure 3:

## 4. Research Results and Discussion

### 4.1. Discussion on Generator Network Structure

In order to solve the problem of DCGAN mode collapse easily, this paper changes the image generator structure into a new structure. As for the processing of the structural mode of the intermediate layer, the author can directly adopt the structural mode of the inverse VGG network and add it to a deconvolution network mode that contains more layers and smaller center displacement of the convolution kernel to directly conduct the data sampling processing and processing on the data of the structural mode of the characteristic layer. The author also here can directly see the step how to use the deconvolution of the network (convolution kernels center displacement of 2) and the structure of the model to directly complete it instead of using network model to deal with the anti-pooling characteristics of the layer structure model of all kinds of data stored on the network sampling. These data can keep all the different types of network information level data on the connection between as well as to the connection between the various network parameters information layer and network layer, and data exchange of information between data acquisition and transmission way will therefore change and become more simple and highly effective and economically efficient. Compared with the anti-pooling network, information loss is much reduced.

### 4.2. Discussion on Discriminator Network Structure

For the use of discriminator network, the author uses two criteria to discriminate: the original image: the original image width and half of the height for correlation discrimination. Model of the feature extraction part of the discriminator network, *H* where: image height, *W*: image width, DF: number of filters.

First, the first convolution group is fused to form a feature graph, and then the 1/2 feature graph is convolved and fused to the original feature graph and then merged into a feature graph and entered into the next volume group. The second method is to directly replace the mean pooling layer, which is less commonly used in the design of convolutional neural network originally designed by the author, with another convolutional layer with a large number of convolution kernels whose step number is more than 2 times, so as to achieve the purpose of next sampling. In this method, in order to effectively achieve more information retention than the original design of directly replacing with the mean pooling layer or replacing with a maximum pooling layer, the author added the InstanceNormalization layer after the convolution layer to better adapt to how to define the loss function. Solve the problem of bad parameters [18].

**Figure 4:**
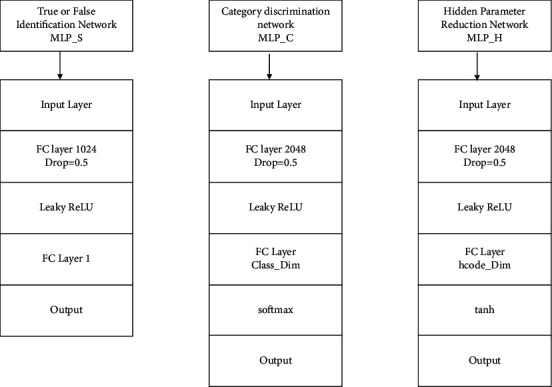
Three fully connected neural networks at the end of the discriminator module.

The following process can be used to identify the convolutional neural network output. Input three MLP-S without shared parameters, which are, respectively, used to distinguish the authenticity of images, identify network types, and restore implicit function parameters. Although these three networks have the same structure, different input layers can be used to define loss function, and their model structure is shown in Figure 4.

### 4.3. Discussion on Loss Function

This model is observed from the perspective of network structure. The output of network structure is composed of three parts. The first part is the image “true or false.” The second part is image category. The third part is the implicit parameter corresponding to the image. In order to solve the problem of mode collapse and the defect of divergence used in the original loss function, Wasserstein JS distance was adopted to represent EarthMover (EM) distance from the real data set to the generated *P*_date_ data set. Therefore, *P*_*g*_ the definition of “true and false” loss function is shown in the following formula:

(4) $$\begin{array}{c} L_{S}=\underset{\text{//}f/{/}_{L\leq 1}}{\text{sup}}E_{X∼P_{\text{data}}}\left[f\left(x\right)\right]-E_{X∼P_{x}}\left[f\left(x\right)\right], \end{array}$$

where *f* is a function we hope to find, indicating //*f*//_*L*≤1_ that the function satisfies Lipschitz-1 continuity; that is, the function is a contraction map [19].

However, since the required function is *f* not easy to find, the gradient penalty approach is chosen to approximate the implementation [20].

For the category loss of the image, since the category of the image in this model is independent of the “true or false” image, which is equivalent to the convolutional neural network part of the discriminator and the fully connected classifier at the end to complete an independent classification task, the cross-entropy loss is used for the classification loss, and the labels of the input and output are encoded with one-hot, and if the *y* output of the MLP-C is represented *y*_*i*_ by *y* the value of each component of *y*^,^ the representation, the category label corresponding to the input image *y*_*i*_^,^ is represented by the value of *m* each component of the total number of categories. The labels of the real image are from the label file (preclassified by chromatic aberration), and the image labels generated by the generator are from the random label vector corresponding to the input generator, and then the classification loss is *L*_*c*_ as shown in the following equation:

(5) $$\begin{array}{c} L_{c}=\frac{1}{m}\sum_{i=1}^{m}\left[y_{i}^{,}\text{log}\left(y_{i}\right)+\left(1-y_{i}^{,}\right)\text{log}\left(1-y_{i}\right)\right]. \end{array}$$

For the added hidden parameters, we aim to add a continuous set of parameters in addition to the discrete category labels that can provide some independent control over the image generation. According to the theory of mutual information, if this set of parameters can play an independent control role in the generation of images by the generator (i.e., not highly coupled with *z* the random noise), then the hidden *h* parameters and the *z* random noise should be independent of each other.

In this case, if there exists an encoder (input image compressed into a set of vectors), then *M* for the image from the generator, the output  *G*(*Z*′) is obtained through *M* the encoder *zσ*_*X*_^2,*∗*^=*M*(*G*(*z*′)), and it should be *h* possible to separate the individual components. If the hidden parameters of the input generator, *h*=(*h*_1_, *h*_2_ …, *h*_*n*_) the solution of the *h*=(*h*_1_^*∗*^, *h*_2_^*∗*^ …, *h*_*n*_^*∗*^) MLP-H output, then we define the reduction loss of the hidden parameters *L*_*h*_ as in

(6) $$\begin{array}{c} L_{h}=\sum_{i=1}^{n}{\left(h_{i}^{*}-h_{i}\right)}^{2}. \end{array}$$

Also, the optimization of the reduction loss of the hidden parameters can reduce the possibility of pattern collapse. We know that pattern collapse occurs when different random vectors are input, and then extremely similar or identical results are output. That *z*_1_^,^ ≠ *z*_2_^,^ is, but *G*(*z*_1_^,^)=*G*(*z*_2_^,^) at this time, *G*(*z*_1_^,^)=*G*(*z*_2_^,^) due to, so after the discriminator feature extraction, but *D*[*G*(*z*_1_^,^)]=*D*[*G*(*z*_2_^,^)] the MLP-H network will try to calculate the part of the reduced random vector according *z*^,^ to *h* the extracted features, at this time, because the function has the feature of one input and only one output, so the calculated result will definitely be *h*_1_^*∗*^=*h*_2_^*∗*^, but the initial input into the generator vector is *h*_1_ ≠ *h*_2_ likely, so the loss of the objective function will be large. Optimization for this part of the objective function will force *G* the generator to *z*^,^ generate different results for different inputs. Although ACGAN also has category loss, the problem of too small a difference between images of the same category still occurs because the categories are discrete variables.

For the three losses defined in the above, *L*_*S*_ and, *L*_*c*_ through *L*_*h*_ experimental observation, we can learn that they are often not in the same order of magnitude numerically, and if the total loss is defined by simply adding up the sums often the small magnitude losses are ignored in the optimization, and at the same time, due to the independent branching structure of the total network, it is not very reasonable to update the parameters of the whole network at one time, so the following parameter update is used in training this model.

- *L*_*S*_ When optimizing the loss, *D* update, there are the network parameters of D-CNN and MLP-S. Update *L*_*c*_ for optimization loss, *G* the network parameters of D-CNN and MLP-S.
- *L*_*h*_ For optimization loss, update *G* the network parameters of D-CNN and MLP-S.

In this way, the parameters of the generator and the feature extraction network are updated 3 times alternately in one update, and the network parameters of each branch are updated once at the same time. The use of gradient penalty to counteract the loss makes it unnecessary for the *L*_*s*_ loss to go through the Sigmoid function, avoids the unreasonable definition of the distance between the generator distribution and the true distribution due to the use of JS scatter, and solves the problem of updating the parameters of the entire discriminator network at one time in the ACGAN model, which also updates the parameters of the optimized category loss. The unreasonableness of the “true and false” discriminant loss is also solved.

### 4.4. Analysis of Experimental Results

#### 4.4.1. Data Set and Labels

In order to further train and complete the method of color matching of original anime characters and the generated character models that will be further introduced in this paper, the authors have collected about 25,000 pixels of original anime characters' avatars from the Internet today, which are from different versions of the original anime characters, and all images are labeled with the same color as them. All images are marked with the same pixel resolution as their respective versions for the capture of the original anime character's facial features. The creators will use the lbpcas-cadeanimefcae script from the open source software to capture and analyze the facial information of the original anime characters from the major gallery sites. In order to achieve the final design goal of the author's anime character color model generation, the model will be mainly designed to be applied to the character avatars on personal information pages, etc. Therefore, this paper needs to be able to uniformly scale and convert all these images into a relatively fixed size image to be used as a network for training to generate this model.

Since all the automatic image generation method models that the authors will discuss in the previous paper must first have an automatic image generation method-assisted model classifier, the authors conclude this paper with the need to reconsider and further refine the approach to automatic image generation method models with centralized processing method functionality based on real image data reclassification. After careful observation, the authors soon found that when the model designers used some relatively new and original versions of WGAN, LSGAN, DCGAN, etc. for image color model generation and postproduction design, the head hair tones often formed a serious degree of color crossover and mingling with the image background color, and the boundaries were not clear. Therefore, in this paper, we will use the head hair color as one of the main categories of the image color expression, so that the modelers can try to learn how to apply the hair color to the “drawing” image by this method to increase the success rate of hair color matching in images.

Based on the large number of basic color data sets that the authors have personally measured in practice and the basic color theory based on CIEDE2000 color difference theory calculations, we are hopeful that we can determine, in practice, the 8 basic color categories that were calculated, which can be referred to as black, red and white, red, green, blue, brown, purple, and yellow, respectively. Through the application of CIEDE2000 color difference theory to analyze through the calculation of the hair color measured by the most should belong to which kind of what type of hair color class to analyze further analysis to further determine the hair image color category, so that we can finally, directly get the hair label.

#### 4.4.2. Generating Model Evaluation

**Figure 5:**
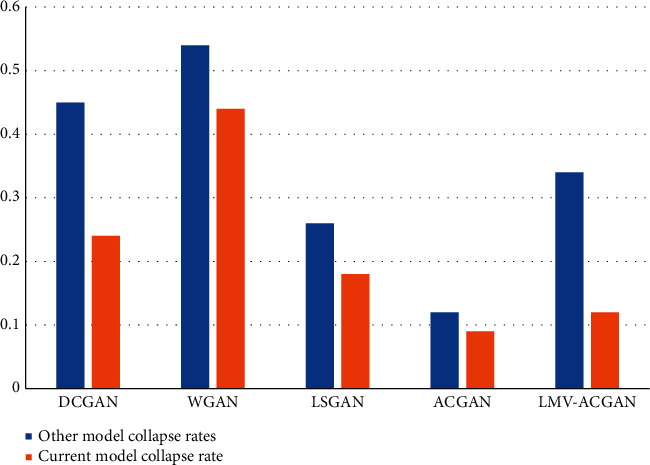
Comparison between other models and the present model collapse rate.

The collapse of the model will mean that the image will look like the real one, but in reality, it is likely to be a real one that looks very much like the impossible one. The model in this paper is a good solution to the problem of model collapse in practice. The generated model solves the problem of collapse better, and the number of collapses decreases significantly. The collapse rates of the models used in this paper have all decreased significantly. The authors have not been able to find out that although these models are generally easy to generate images accurately and quickly but normally and effectively in the early stages of the development of the training generation models; in fact, after the models have reached the level of 100th generation of the training generation (epoch), the DCGAN models and WGAN models are very likely or effective. However, in fact, after the training generation (epoch) of the model has reached the level of 100th generation, the DCGAN model and the WGAN model are very likely or easy to have a serious error result of image pattern collapse. In contrast, the model in this paper has completely solved the problem of model collapse and improved the image realism and color matching of anime characters only at a certain level of theoretical foundation [20] as shown in Figure 5.

**Figure 6:**
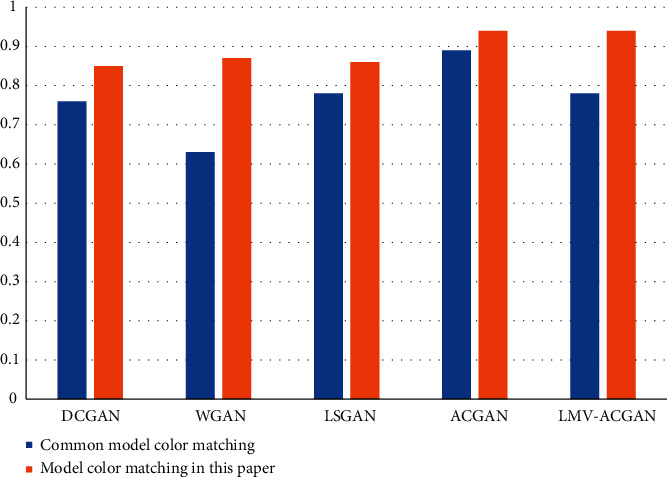
Comparison of color matching degree of anime characters.

The higher the value, the higher the color matching of the anime character, and the better the color matching of the anime character designed in this paper. This index is an indirect index. After comparison, it is found that the color matching degree is significantly improved in this design model, as shown in Figure 6.

**Figure 7:**
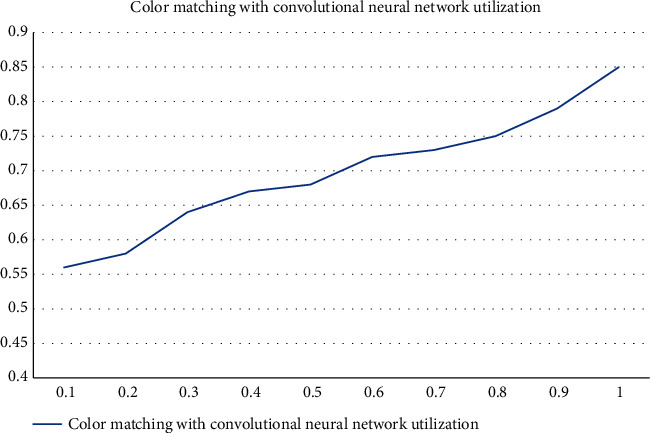
Color matching with convolutional neural network utilization.

This algorithm study also investigated the relationship between the color matching degree of the anime character and the utilization rate of the convolutional neural network algorithm, and the horizontal coordinate of Figure 7 is the utilization rate of the convolutional neural network algorithm, and the vertical axis is the color matching degree. The results show that as the utilization rate of convolutional neural network algorithm keeps improving, the level of color matching of anime characters also keeps improving. Although this study investigated the relationship between the color matching degree of anime characters and the utilization of convolutional neural network algorithm, there is a significant effect between the two. However, this study is an indirect study and does not directly prove a correlation. Even if there is a regression relationship, it is necessary to include other variables for comparison and to consider the influence of disturbing factors, to consider the statistical relationship comprehensively, and to consider the range of *p*-values as well as the range of u-values as shown in Figure 7.

### 4.5. Discussion of Image Generation by Category

In this paper, the model design only supports the automatic image generation under the specified category conditions. In order to make the analysis and comparison between the further model design and the modification of the original ACGAN model, the authors have recently modified the system and optimized the design and improved the system by using the same method as the original ACGAN model. The authors have recently redesigned and optimized the system to use the automatic image generator and the automatic image performance discriminator and analyzer that may be used in the modification of the original ACGAN model.

For CGAN, in addition to the above-mentioned differences between the same type of ACGAN tags, one of the most important features of CGAN tags is that they can be used as part of the network image feature extraction by direct input, together with the network image tag part and the final discriminator part of the direct input method. The final discriminator part is generally responsible for inputting the network image features as true or false, but not for outputting the network image features to be extracted from the input, and can then perform a new network image classification based on the additional network image features provided in the network input information to obtain another classification of the network label, which can be used to compare with the input network label features. The network labels are then used for analysis and comparison with the network label features of the input information. The authors have now trained and tested these three models for automatic image generation by category after completing about 300 epochs. After further careful study and analysis, the authors further found that the three original models mentioned above have been or can be fully implemented to identify and generate each category of image errors in the input images and to separately identify and generate each category of image errors in the input images according to the requirements. For the original model of CGAN, similar to the original model of ACGAN, there are several phenomena that may occur at the same time when there are errors in recognition or when recognition fails to generate when there are errors in individual input images or multiple image categories. In the process of recognizing the color of multiple input image categories, there are several common situations in which the color discrimination may be wrong (e.g., the color discrimination of white and brown categories of CGAN is wrong or cannot be generated, the blue category of ACGAN cannot be generated correctly, and the green and brown categories are wrongly judged by the model), and special optimization processes are carried out for such special situations.

### 4.6.  The  Role of the Implicit Function in Generating the Model

In this section, the noise parameter vector *z* and the image class parameter vector *c* are introduced for the special treatment of the images, and the process requires special attention in the next steps to make them as stable as possible when observing all the images in the image dimension and any values in the other or in any other range of image dimensions and to keep the maximum value at or equal to or less than to 0. In this experimental and research process model, the authors will also be able to observe and obtain, at the same time, that the continuous variation of each image dimension value parameter will be such that one image parameter in the image generated by the authors' research will be able to produce a continuous variation in each or several image dimensions, while it will also vary continuously with each other, such that it will be able to produce a continuous variation in each image dimension parameter. The continuous changes of the image parameters in each dimension are also independent of each other, and the continuous changes of the image parameters in each dimension can affect the continuous generation process of the whole image parameters. The main feature of the 1st dimensional parameter that can be used to control the change and transition of the image is simply the relative value of the length of the hair, which is obviously a transition from a medium-length image with a value of -1 or so to a medium-length image with a value of 1 or less that can be directly observed. The transition from a medium-length image with a value of about -1 or less to a medium-length image with a value of about 1 or less and the whole process of the transition in this mutual continuous change will be a mutual continuous change in the transition process; observing the 2nd dimensional hair data, it is already obvious that the hair parameter here is mainly used to control another hair parameter that can be used to control the facial features of the hair, that is, the shade value of the hair color, from a value of less than -1 or an absolute value greater than 1 when the overall color of the hair is light dark red. When the value is 0 or the value is equal to 1, the overall color of the hair is dark. By comparing the 3rd and 4th dimensional observations, it is possible to obtain the intuitive result that these two data have the property of being transformed into each other; comparing the observation data in the 5th dimension, the author will also be able to see a more intuitive and clearer comparison of the observation data and will find that the color matching effect is very good. In the process of controlling the generation of the actual parameters of the image, the authors may also or may not be able to simultaneously consider how the generation of the “detail” parameters of the image can be controlled by modifying some of the hidden actual parameters.

### 4.7. The Role of Hidden Parameters and Multiscale Discriminations in Training and the Impact on the Auxiliary Discriminators

**Figure 8:**
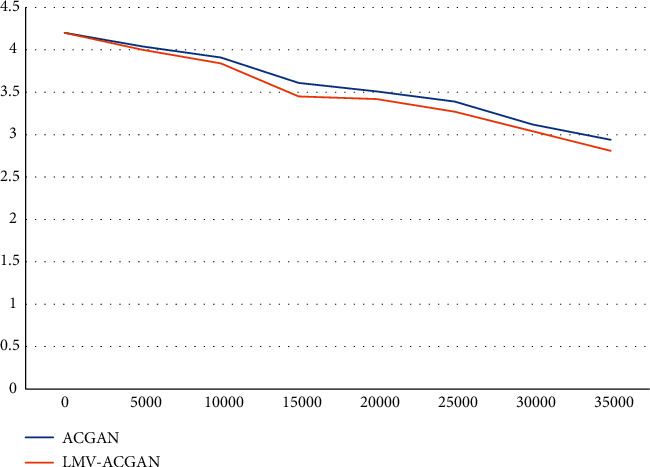
Comparison of classification loss of ACGAN and LMV-ACGAN.

A comparative analysis and illustration are presented to show how the ACGAN model is used in the original early version and the LMV-ACGAN model presented in this paper and how the loss values of the auxiliary classifier parameters change during the actual training process. From the comparison of the graphical results, it is clear to the authors that the number of losses of the implicit parameters in the model is increasing gradually and rapidly and that the discrimination and errors that may arise in the multi-implicit parameter scale model are clearly likely to develop further, leading to an increase in the number of losses in the parameters of the scale model. However, I have not seen any direct impact on the convergence rate of the model parameters due to the continuous and rapid increase in the amount of hidden parameter losses. At the same time, the weighted mean value of the classification loss information is obtained after increasing the number of iterations to about 35,000 per second; that is, the authors can directly see that the weighted mean value of the classification loss information obtained after increasing the number of iterations to about 35,000 per second has been significantly reduced from the original 2.94 to nearly half of the current value and has now significantly exceeded the original 2.81, by a certain degree. This is undoubtedly a significant increase in the accuracy of the image classification function and the image judgment ability of the auxiliary image generator when generating auxiliary images by category. At the same time, this model can ensure faster and more effective image classification and discrimination results at the performance level with fewer iterations and generation times than the model that can be applied to ACGAN independently as shown in Figure 8.

## 5. Conclusion

Animation media language refers to a kind of audio-visual combined with a comprehensive use of audio-visual effect of the animation language, rapid and convenient mode of transmission, and young and old children of both sexes, which also often contains the performance of the integration of a variety of traditional human values, outlook on life, and world view, reflecting a profound and rich variety of national cultural significance and ideological tendencies enough to affect deeply the daily work of people in contemporary society. It is a correct choice of various social ways of thinking and behavior concepts and various social life path patterns. Anime media is one of the most important media that the author has vigorously inherited and developed under the essence of our long-standing Chinese traditional animation culture. Anime character modeling design is one of the most fundamental cores of the author's styling creativity for all traditional animation works. The content of anime character modeling design usually also includes traditional anime character image modeling design, character color modeling design, costume style design, and action modeling design content. The color part is also one of the main visual symbols to show the most direct and vivid and intuitive character effect in anime, and the design techniques of color element matching and color generation have been occupying an extremely important position in almost all the visual design methods of anime character modeling.

In this paper, we innovatively used convolutional neural network algorithm to innovate the color matching generation of anime characters and improve the traditional technology of color matching of anime characters. This paper mainly utilizes Generative Adversarial Network (GAN), Deep Convolutional Generative Adversarial Network and VGG model, and multiscale discriminator theory, uses ACGAN research method and this paper's innovative LMV-ACGAN research method for study, and concludes that other models have higher collapse rate than this model; this model has higher color matching of anime characters, and color matching improves with the utilization of convolutional neural network improved with the increase of convolutional neural network utilization and other conclusions. Moreover, the advantages and disadvantages of this study are reviewed, so that later researchers can have a more rational and objective understanding of this study. In general, this model has advantages over the traditional model, but there are problems such as pseudoregression that needs the attention of later researchers.

## Data Availability

The dataset can be accessed upon request.
